# Statistical Analysis and Preliminary Study on the Mix Proportion Design of Self-Compacting Steel Fiber Reinforced Concrete

**DOI:** 10.3390/ma12040637

**Published:** 2019-02-20

**Authors:** Xinxin Ding, Minglei Zhao, Siyi Zhou, Yan Fu, Changyong Li

**Affiliations:** 1 International Joint Research Lab for Eco-building Materials and Engineering of Henan, North China University of Water Resources and Electric Power, Zhengzhou 450045, China; syzhou@stu.ncwu.edu.cn (S.Z.); yanfu@stu.ncwu.edu.cn (Y.F.); lichang@ncwu.edu.cn (C.L.); 2 School of Engineering, RMIT University, Melbourne, VIC 3001, Australia

**Keywords:** self-compacting steel fiber reinforced concrete (SC-SFRC), mix proportion, fiber factor, water-to-binder ratio (*w*/*b*), cubic compressive strength, splitting tensile strength, calculation model

## Abstract

With the sustainable development of green construction materials in civil engineering, self-compacting steel fiber reinforced concrete (SC-SFRC) has attracted widespread attention due to its superior self-compacting performance and excellent hardened properties. In this paper, 301 groups of test data from published literatures were collected to quantify the characteristics of the mix proportion of SC-SFRC. The type, aspect ratio and volume fraction of steel fiber commonly used in SC-SFRC are discussed and the effects of steel fiber on the workability and mechanical properties of SC-SFRC are statistically studied. The relationship of cubic compressive strength and water-to-binder ratio and that of the splitting tensile strengths between SC-SFRC and referenced self-compacting concrete (SCC) are also evaluated. Based on these analyses, the reasonable ranges of material components in the mix proportion design of SC-SFRC are determined. The results showed that with several adjusted parameters, the calculation model of the water-to-binder ratio for the mix proportion design of ordinary concrete is suitable for SC-SFRC. The calculation model of tensile strength is suggested for SC-SFRC with various types of steel fiber.

## 1. Introduction

Self-compacting steel fiber reinforced concrete (SC-SFRC) is a type of high performance concrete with the benefits of self-compacting performance at the fresh stage and superior mechanical properties at the hardened stage. The composition, production and mechanism of SC-SFRC are more complicated than those of self-compacting concrete (SCC). Due to the large density, elongated shape and large surface area of steel fiber [1], the direct addition of steel fibers in SCC would impair the self-compacting performance and disturb the solid skeleton of fresh concrete [2,3,4]. Nowadays, studies showed that a limited volume fraction of steel fiber could be used in SC-SFRC in order to ensure higher workability and better mechanical performance. Due to the packing effect of the fiber-aggregate solid skeleton, the self-compacting performance could not be achieved once the volume fraction of steel fiber in concrete exceeds the limited value even if the concrete mixture is a homogeneous and stable suspension [5]. The limitation (usually less than 2%) is mainly affected by the characteristics of raw materials [2,6,7] and mix proportion [8,9,10,11,12]. The characteristics of the raw materials include the maximum particle size and surface morphology of coarse aggregate, volume fraction, aspect ratio and geometry of steel fiber, binder paste content and sand ratio. The volume fraction of steel fiber used in SC-SFRC increased in proportion to the sand ratio [12]. A higher volume fraction of steel fiber for SC-SFRC could be used by altering the compositions of concrete mixture and adjusting the characteristics of steel fibers. 

At present, various mix design methods for SC-SFRC have been reported. The binary mixture method [4,9,13,14,15,16,17,18,19,20,21] is one of the most commonly used methods, in which steel fibers are directly added to SCC. The workability of fresh SC-SFRC is tested to evaluate its satisfaction in terms of the required self-compacting performance. If self-compacting could not be achieved, the material compositions were subsequently adjusted without affecting the water-to-binder ratio (*w*/*b*). The adjustment measures include adding chemical admixture [13,18,19,20,21] and taking the volume of steel fiber into the consideration of an aggregate [9,15,16] or coarse aggregate [4]. The method has several disadvantages, such as project targeting, particularized condition and poor adoptability. The actual applications relied on the experience of engineering technicians.

Another mix design method is the modified Densified Mixture Design Algorithm (DMDA) [22,23], which is based on the required durability. The principle is low water content and low cement content to enhance the durability. The detailed measures included three steps. Firstly, we maximized the pile-up density of aggregates and fly ash using the loose packing test to optimize the content of fly ash and aggregates. After this, we added steel fibers as part of the solid volume and calculated the lowest void fraction of solid skeleton. Finally, we calculated the total paste volume with consideration of the thickness of lubricating paste. It can be seen that only the influence of volume of steel fiber is taken into account in this method.

Grünewald [2] proposed a performance-based mix design method for SC-SFRC. This method uses the Compressive Packing Model (CPM) [3] and assumes that the steel fiber is the equivalent packing diameter [24]. The virtual packing density of the steel fibers was calculated from the experimental packing density under the consideration of the wall-effect (experimental results) and the applied compaction process (the compaction index K = 3.6, which is experimentally determined for the deposition method). Ferrara [25] presented a “rheology of paste model” mix design method for SC-SFRC. The method chose to include steel fibers in the particle size distribution of the solid skeleton by creating the concept of an equivalent diameter of the same specific surface. The parameters of these methods have complex calculation processes and require high theoretical knowledge for engineering technicians.

Eduardo [26] and Barros [7] produced SC-SFRC by optimizing the composition of binder materials and that of aggregates, respectively. The optimization of binder materials is based on the requirements of both workability and compressive strength. The optimization of the particle size distribution of aggregates is experimentally determined by the packing test of steel fiber–aggregates solid skeleton. In addition, the volume fraction of steel fiber is included according to the engineering requirement. The minimum binder paste in SC-SFRC is chosen according to the self-compacting performance. This method is project targeted and needed a large amount of experimental data to evaluate the influence of steel fiber on the solid skeleton.

Besides, Sahmaran [27] attempted to produce SC-SFRC by simply increasing water content with constant contents of binder and chemical admixtures. Cai [28] produced SC-SFRC by increasing contents of water and binder with constant *w*/*b*. Anastasiou [29] produced SC-SFRC by increasing the amount of ladle furnace slag used with constant *w*/*b*. Li [12] reported that the optimal sand ratio of SC-SFRC should consider the volume fraction of steel fibers.

The solid skeleton of aggregates or steel fiber–aggregates and its balance with the binder content are considered in the above-described mix design methods. The addition method of steel fibers is the major difference among them. The limit of the type and volume fraction of steel fibers in these methods are not clear. Thus, more statistical analysis, quantity trails of experiments and a simplified calculation process are needed for determining the mix proportion design of SC-SFRC.

In this paper, the experimental database of SC-SFRC is built. Combined with the previous study of the numerical analysis of mix proportion for SCC [30], the differences of compositions between the SC-SFRC and SCC are systemically discussed. Moreover, the type, aspect ratio and volume fraction of steel fibers commonly used in SC-SFRC are discussed. The effects of the fiber factor on the workability and mechanical properties are statistically studied. The relationship of *w*/*b* and cubic compressive strength of SC-SFRC as well as splitting tensile strengths between SC-SFRC and referenced SCC are studied.

## 2. Statistical Analysis for Mix Proportion of SC-SFRC

### 2.1. Experimental Database

In this paper, 301 groups of test data for SC-SFRC from previous literatures [2,9,10,12,13,14,15,16,17,18,19,22,23,26,27,28,29,31,32,33,34,35,36,37,38,39,40,41,42,43,44,45,46,47,48,49,50,51,52,53,54,55,56,57,58,59,60,61] were collected to build the database. The range of results used for the experimental database are listed in Table 1. The mineral admixtures of SC-SFRC in the experimental database included parts of fly ash, slag powder, silica fume and stone powder. It should be noted that some values of the cylinder compressive strength used in the experimental database were converted into the cubic compressive strength (*f*_cu_). The conversion factors are followed by the reference [62]. The detailed information of experimental database for SC-SFRC is presented in Appendix A. 

### 2.2. Aggregates

**Figure 1:**
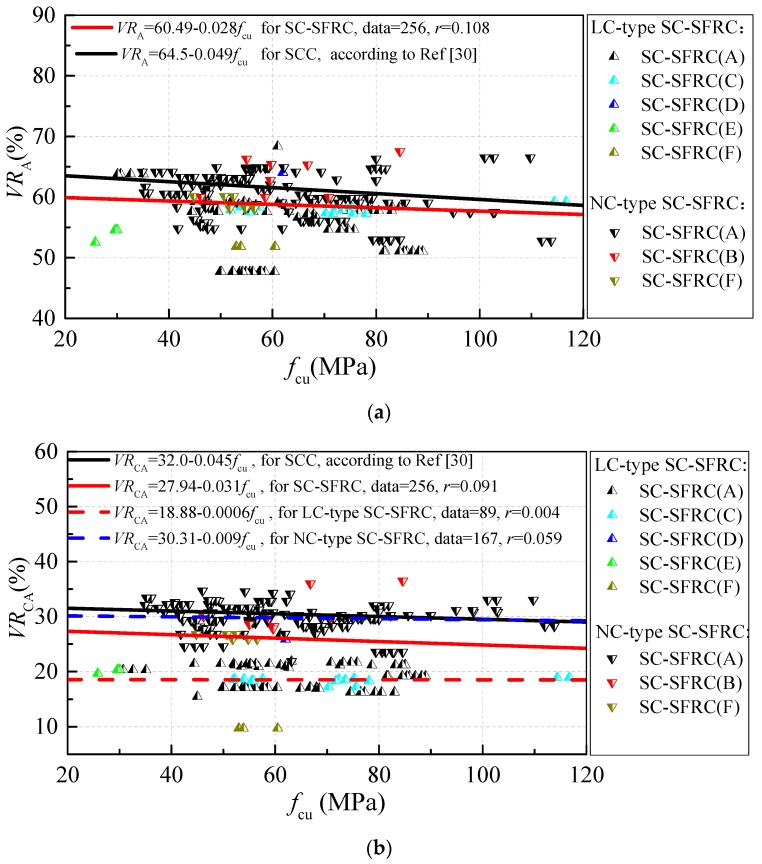

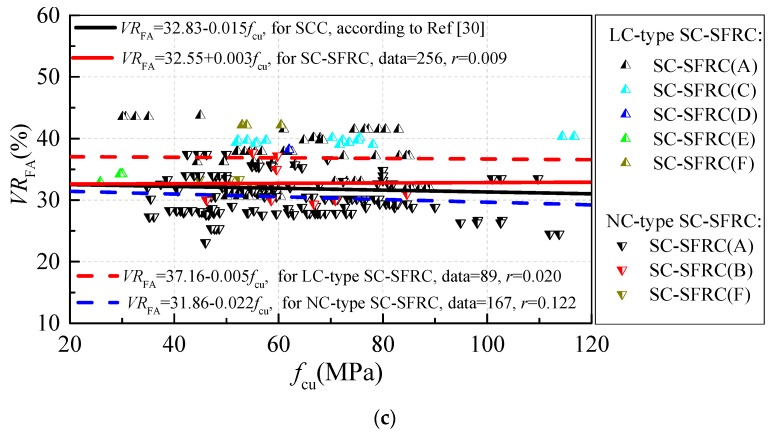
The differences of volume fraction of aggregates: (**a**) *VR*_A_; (**b**) *VR*_CA_; and (**c**) *VR*_FA._

Figure 1 displays the variations of the volume content of aggregates, coarse aggregate and fine aggregate (*VR*_A_, *VR*_CA_ and *VR*_FA_) along with the cubic compressive strength *f*_cu_ for SC-SFRC. SC-SFRCs (A)–(G) form the test data of SC-SFRC with a binary mixture design method, modified DMDA method, modified CPM method, method based on the packing test of steel fiber–aggregates, method of changing water content, method of increasing water and binder contents with *w*/*b* constant and method of increasing binder content, respectively. The volume content of their raw materials are distinguished using points of different color. *VR*_a_, *VR*_CA_ and *VR*_FA_ changed by 45–70%, 10–35% and 25–45% for SC-SFRC, respectively. Both *VR*_A_ and *VR*_CA_ show a small decrease with increased cubic compressive strength. *VR*_FA_ shows little regularity with increased cubic compressive strength. Linear fitting was used to obtain the trendline. Trendlines of *VR*_A_, *VR*_CA_ and *VR*_FA_ along with *f*_cu_ for SCC [30] are drawn as black lines where *r* is the correlation coefficient of the regression equation. It can be seen that *VR*_A_ and *VR*_CA_ of SC-SFRC are about 1.49–3.59% and 4.18–4.78% lower than those of SCC, respectively. Compared with SCC, one feature of the mix proportion for SC-SFRC is the lower *VR*_A_ and *VR*_CA_ values.

**Figure 2:**
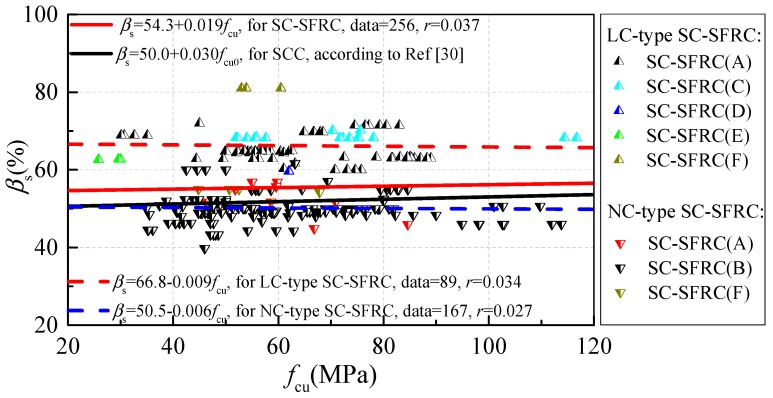
Variation of sand ratio *β*_s_ along with cubic compressive strength *f*_cu._

In term of self-compacting performance, a smaller content of coarse aggregate leads to less blocking effect and high flowability of fresh concrete. A suitable volume content of fine aggregate and a certain range of sand ratio would increase the segregation resistance of fresh concrete. However, a higher volume content of coarse aggregate would be beneficial in restricting the shrinkage deformation of hardened concrete, which is consistent with the requirements of the concrete structure performance. Thus, considering the different volume contents of coarse aggregate and fine aggregate, the mix proportion of SC-SFRC is classified into two types, which was named as NC-type SC-SFRC (legends of chamfering triangle in Figure 1 and Figure 2) and LC-type SC-SFRC (legends of triangle in Figure 1 and Figure 2), respectively. NC-type SC-SFRC has a volume content of coarse aggregate that is more than 600 kg/m^3^ and volume content of fine aggregate that is less than 1000 kg/m^3^. LC-type SC-SFRC has coarse aggregate content that is less than 600 kg/m^3^ and fine aggregate content that is more than 1000 kg/m^3^. LC-type SC-SFRC focuses more on the performance of fresh concrete, while NC-type SC-SFRC focuses on both the workability and volume deformation of hardened concrete. Similar *VR*_A_ values were observed in NC-type and LC-type SC-SFRC. LC-type SC-SFRC has a *VR*_CA_ and *VR*_FA_ that was lower by 10.75–11.59% and higher by 5.64–7.34%, respectively, than NC-type SC-SFRC. 

*VR*_A_, *VR*_CA_ and *VR*_FA_ of SC-SFRC with different mix design methods also showed some differences. SC-SFRC (C), SC-SFRC (D), SC-SFRC (E) and part of SC-SFRC (A) and SC-SFRC (F) can be categorized to LC-type SC-SFRC. In contrast, SC-SFRC (B) and part of SC-SFRC (A) and SC-SFRC (F) can be categorized to NC-type SC-SFRC.

Figure 2 shows the variation of sand ratios according to mass *β*_s_ along with the cubic compressive strength. *β*_s_ changed by 40–80% for SC-SFRC, which is about 3.0–4.1% higher than that of SCC. Moreover, more than 80% of the sand ratios of SC-SFRC were 47–69%. *β*_s_ of LC-type SC-SFRC was higher by 15.9–16.2% than that of NC-type SC-SFRC.

**Figure 3:**
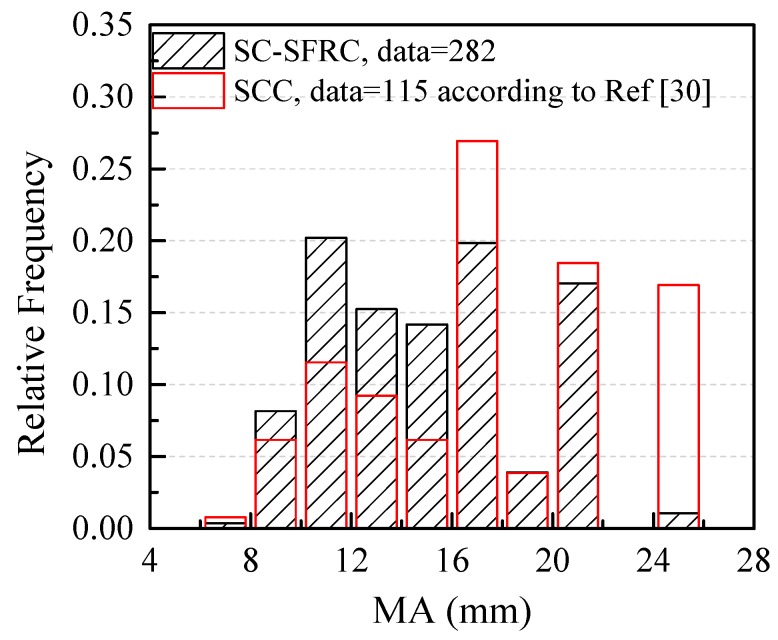
The probability distribution of maximum particle size of coarse aggregates.

Figure 3 shows the probability distributions of the maximum particle size (MA) of coarse aggregates for SC-SFRC and SCC. The test data of SC-SFRC with MA ≤ 10 mm, 10 mm < MA ≤ 16 mm, 16 mm < MA ≤ 20 mm and 20 mm < MA ≤ 25 mm are 28.4%, 49.6%, 21.0% and 1.0%, respectively. The test data of SCC [30] with MA ≤ 10 mm, 10 mm < MA ≤ 16 mm, 16 mm < MA ≤ 20 mm and 20 mm < MA ≤ 25 mm are 18.5%, 42.3%, 21.5% and 16.2%, respectively. Thus, one feature of SC-SFRC is the smaller MA of coarse aggregates compared to SCC. 

### 2.3. Binder and Binder Paste

**Figure 4:**
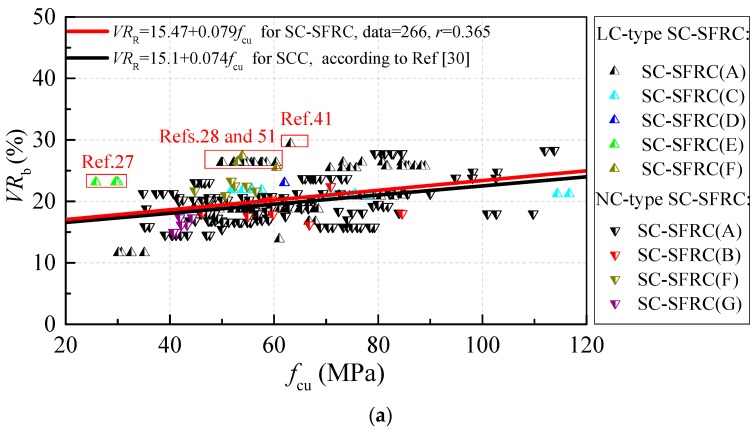

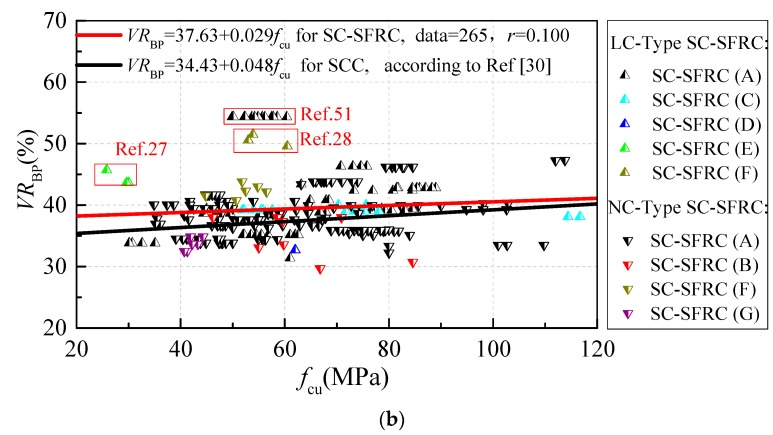
The differences of binder materials: (**a**) *VR*_b_; and (**b**) *VR*_BP_.

Figure 4 exhibits the variations of volume contents of the binder and binder paste (*VR*_b_, *VR*_BP_) along with the cubic compressive strength *f*_cu_. Similar to Figure 1 and Figure 2, the legends of the chamfering triangle are NC-type SC-SFRC and the legends of triangle are LC-type SC-SFRC. In this paper, the binder material is the sum of cement and mineral admixtures while *VR*_BP_ is the sum of water and binder materials by volume. The influence of hydration reaction on the volume change of binder paste is ignored. *VR*_b_ and *VR*_BP_ changed by 12–30% and 30–55% for SC-SFRC, respectively. Similar to SCC [30], *VR*_b_ and *VR*_BP_ of SC-SFRC increased in proportion to the cubic compressive strength. *VR*_R_ and *VR*_BP_ of SC-SFRC are higher by approximately 0.47–0.97% and 0.92–2.82% compared to that of SCC, respectively. Thus, one feature of the mix proportion for SC-SFRC is the higher *VR*_BP_.

As shown in the red box line of Figure 4, several test data have higher *VR*_b_ and *VR*_BP_ values [27,28,51]. All these test data belong to LC-type SC-SFRC. Moreover, the experimental study of Abrishambaf used abundant binder materials of 766 kg/m^3^ and a slightly low water content of 140 kg/m^3^ [41] with *f*_cu_ = 63 MPa. The limestone filler was considered as one of the binder materials. 

*VR*_b_ and *VR*_BP_ of SC-SFRC with different mix design methods also show some differences. Comparing with the trendlines of *VR*_b_ and *VR*_BP_, SC-SFRC (C) and SC-SFRC (G) have similar *VR*_b_ and *VR*_BP_, SC-SFRC (E) and part of SC-SFRC (F) have higher *VR*_b_ and *VR*_BP_ due to the same reason discussed in the above paragraph. In contrast, SC-SFRC (B) and SC-SFRC (D) have lower *VR*_BP_ due to the lower water content.

### 2.4. Steel Fiber

It can be summarized from the experimental database that the hooked-end steel fiber is the most commonly used, which was involved in about 54% of SC-SFRC. This was followed by 23% of SC-SFRC containing crimped steel fibers. All of the other macro steel fibers of straight, milled cut, indentation, twisted and large-end account for about 20%. In contrast, micro steel fiber is only involved in about 3% of SC-SFRC.

**Figure 5:**
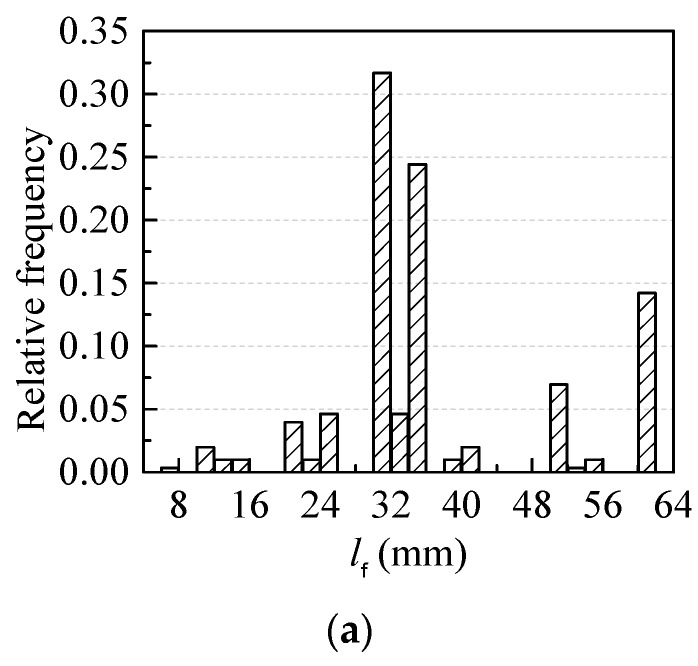

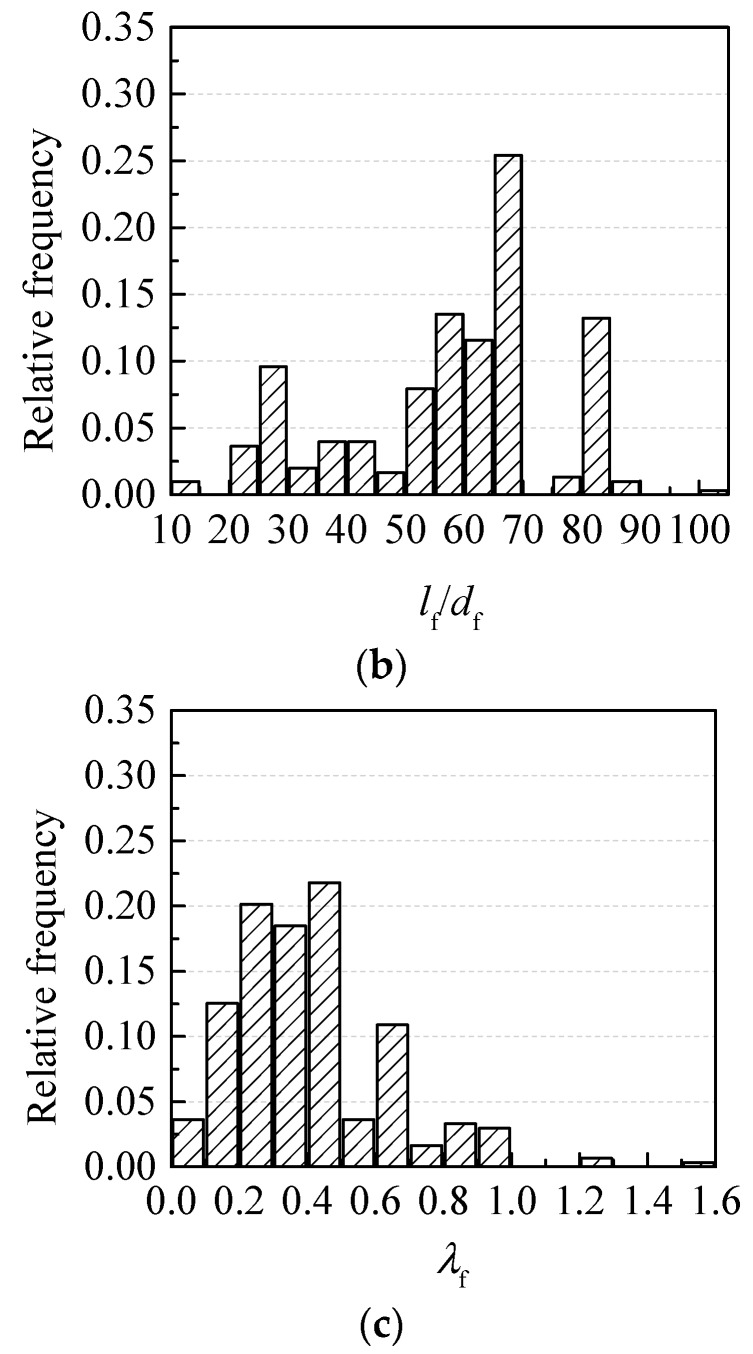
Probability distribution of steel fiber in SC-SFRC: (**a**) length *l*_f_; (**b**) aspect ratio *l*_f_/*d*_f_; and (**c**) fiber factor *λ*_f_.

The histograms of the probability distributions of length *l*_f_, aspect ratio *l*_f_/*d*_f_ and fiber factor *λ*_f_ = *ρ*_f_·*l*_f_/*d*_f_ of steel fiber are drawn in Figure 5 where *d*_f_ is the equivalent diameter of the cross-section of steel fiber and *ρ*_f_ is the volume fraction of steel fiber in concrete. More than 75% of *l*_f_ were less than 42.5 mm, more than 56% of fiber length *l*_f_ ranged from 30 mm to 35 mm, more than 58% of aspect ratio *l*_f_/*d*_f_ ranged from 50 to 70 and more than 80% of the fiber factor *λ*_f_ were less than 0.55.

**Figure 6:**
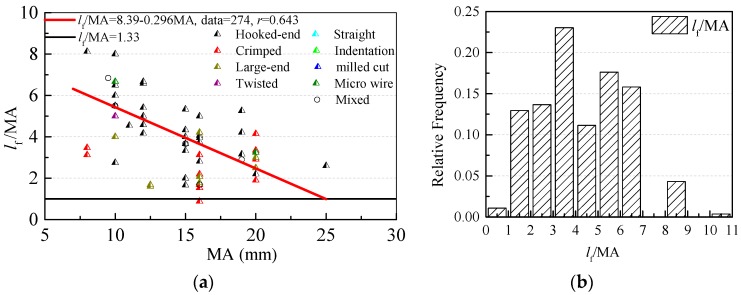
The relationship of fiber length *l*_f_ and the maximum particle size of aggregates MA: (**a**) Variation of *l*_f_/MA along with MA; and (**b**) Probability distribution of *l*_f_/MA.

Figure 6 shows the relationship between fiber length *l*_f_ and the maximum particle size of aggregate MA. To ensure an efficient reinforced effect of steel fibers, *l*_f_/MA should be larger than 1.33 in vibrated-compacting SFRC [63]. It can be seen that 99% of *l*_f_/MA are larger than 1.33 in SC-SFRC. *l*_f_/MA decreases with the increasing MA and 94% of *l*_f_/MA are between 1.5 and 6.5.

**Figure 7:**
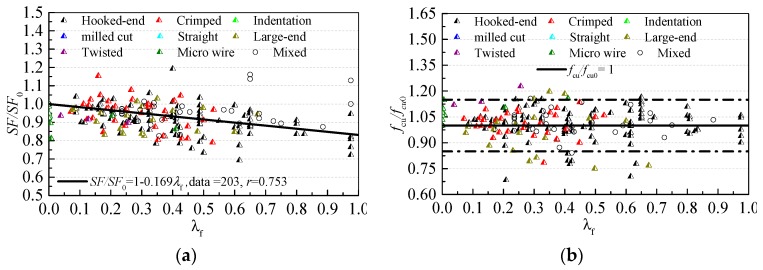
The influences of steel fiber on the fresh and hardened performances of SC-SFRC: (**a**) variation of slump flow ratio SF/SF_0_ with fiber factor *λ*_f_; and (**b**) variation of cubic compressive strength ratio *f*_cu_/*f*_cu0_ with fiber factor *λ*_f_.

The influences of steel fiber on the fresh and hardened performances of SC-SFRC are shown in Figure 7. SF and SF_0_ are the slump flows of SC-SFRC and the referenced SCC, respectively. The ratio of the slump flow SF/SF_0_ forms a linear correlation with fiber factor *λ*_f_, which decreases with an increase in *λ*_f_. The type of steel fiber has no obvious influence on SF/SF_0_. Both the fiber factor and type have no obvious influence on the compressive strength. In this study, *f*_cu_ and *f*_cu0_ are the cubic compressive strengths of SC-SFRC and the referenced SCC, respectively. We determined that 89.4% of the ratios of cubic compressive strength *f*_cu_/*f*_cu0_ ranged from 0.85 to 1.15. 

## 3. Calculation Model of Water-to-Binder Ratio for SC-SFRC

The calculation model of *w*/*b* for the vibrated-compacting concrete in Chinese standard JGJ/T 55 [64] is:

(1) $$w/b=\frac{\alpha_{a}f_{b}}{f_{cu,0}+\alpha_{a}\alpha_{b}f_{b}}$$

where *f*_cu,0_ is the designed cubic compressive strength of concrete; *α*_a_ and *α*_b_ are coefficients mainly related to the type of coarse aggregate; and *f*_b_ is the compressive strength of binder materials at 28 days (MPa), which can be predicted by Formula (2), when there is no measured value.

(2) $$f_{b}=\gamma_{f}\gamma_{s}f_{ce}$$

where *f*_ce_ is the compressive strength of cement at 28 days; *γ*_f_ is the influence coefficient of fly ash, which can be taken as values listed in Table 2 [65]; and *γ*_s_ is the influence coefficient of slag powder, which can be taken as the values specified in JGJ/T 55 [64]. With a similar influence, stone powder has the same influence coefficient with fly ash. Moreover, the influence coefficient of silica fume is taken as 1.0 conservatively in the following sections.

Besides, the calculation model of *w*/*b* for SCC in Chinese Standard JGJ/T 283 [66] is;

(3) $$w/b=\frac{0.42f_{ce}\left(1-\beta +\beta \cdot \gamma \right)}{f_{cu,0}+1.2}$$

where *β* is the replacement ratio by the mass of mineral admixture in binder materials; and *γ* is the influence coefficient of mineral admixtures. These values are 0.4 and 0.9 for fly ash with *β* ≤ 0.3 and slag powder with *β* ≤ 0.4, respectively.

In fact, both Equations (1) and (3) have a form of reciprocal function and are identical in essence, which could be expressed in one form:

(4) $$w/b=\frac{\alpha_{a}\kappa f_{ce}}{f_{cu,0}+\alpha_{a}\alpha_{b}\kappa f_{ce}}$$

where *κ* is the influence coefficient of mineral admixture on the compressive strength of cement. *Κ* = *γ*_f_·*γ*_s_ in JGJ/T 55, *κ* = 1 − *β* + *β*·*γ* in JGJ/T 283.

If *f*_b_ = *κf*_ce_, *α*_ab_ = −*α*_a_*α*_b_, Equation (4) can be transformed to Equation (5).

(5) $$\frac{f_{cu,0}}{f_{b}}=\alpha_{a}\frac{b}{w}+\alpha_{ab}$$

**Figure 8:**
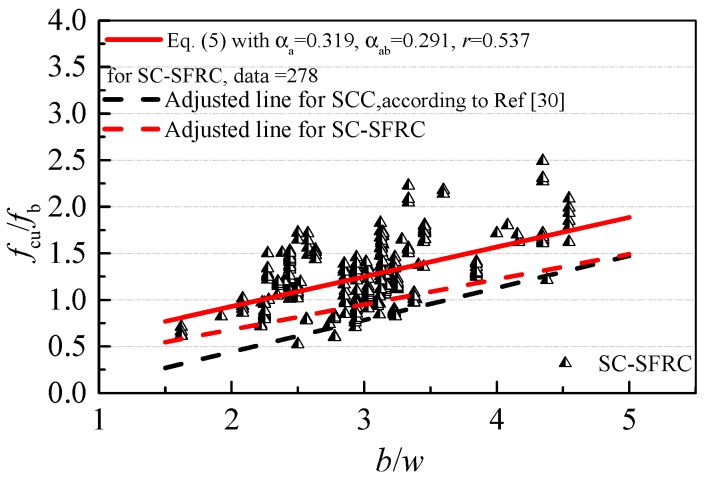
The fitting process of *α*_a_ and *α*_ab_.

With *b*/*w* as the x-axis and *f*_cu_/*f*_b_ as the y-axis, Figure 8 plots the test data and fitting result of SC-SFRC. The parameters *α*_a_ = 0.319 with the standard deviation of 0.030 and *α*_ab_ = 0.291 with the standard deviation of 0.091 are obtained.

Considering the reliability of cubic compressive strength with a probability of 95% in the mix proportion design, *α*_a_ = 0.270 and *α*_ab_ = 0.141 for SC-SFRC are obtained. Thus, *α*_b_ = -*α*_ab_/*α*_a_ = −0.522.

The different coefficients reflect the different changes in *w*/*b* with cubic compressive strength *f*_cu_. With the same *w*/*b*, *f*_cu_ of SCC is about 7–10 MPa lower than that of vibrated-compacting concrete [30]. For SC-SFRC, a *w*/*b* lower than that of vibrated-compacting concrete and higher than that of SCC is needed to achieve the same *f*_cu_. Thus, Equation (1) with *α*_a_ = 0.270 and *α*_b_ = −0.522 is advised for the mix proportion design of SC-SFRC.

## 4. Designed Tensile Strength of SC-SFRC

Steel fibers obviously strengthen the splitting tensile strength in SC-SFRC [20,60]. The calculation model of designed tensile strength *f*_ft_ for SC-SFRC is shown as Equation (6) [60].

(6) $$f_{ft}=f_{t}\left(1+\alpha_{tb}\alpha_{te}\lambda_{f}\right)$$

where *f*_t_ is the tensile strength of referenced SCC, which could be accurately estimated by using the EC-2 model [67,68,69]; *α*_te_ is the coefficient related to the effective fiber distribution; and *α*_tb_ is a coefficient that colligated the other factors, which influence the bridging effects of steel fibers in the splitting tensile test.

A total of 89 groups of splitting tensile test data in the experimental database were used to verify Equation (6). There were different advised values of *α*_tb_*α*_te_ for different types of steel fibers according to Chinese standard JGT 472 [63]. The tested splitting tensile strength *f*_t_ of SCC in the same strength grade was used to calculate the tensile strength *f*_ft,c_ of SC-SFRC. Apart from *α*_tb_*α*_te_ = 0.5, the SC-SFRC with crimped steel fiber and cubic compressive strength of the referenced SCC ranged from 20 MPa to 60 MPa [60]. Moreover, a simple Formula (7) was used to calculate the tensile strength of SC-SFRC with hybrid steel fibers based on the previous study of concrete with hybrid steel fibers [70].

(7) $$f_{ft}=f_{t}\left(1+\kappa_{t}\sum \alpha_{tb}\alpha_{te}\lambda_{f}\right)$$

where *κ*_t_ is a coefficient of the hybrid effect for concrete with hybrid steel fibers. For concrete with hybrid steel fibers, *κ*_t_ > 1 means a positive hybrid effect between steel fibers while *κ*_t_ < 1 means a negative hybrid effect between steel fibers. For SC-SFRC mixed with hooked-end and straight steel fibers in this study, *κ*_t_ ≈ 1. It means that the hybrid effect did not exist in the hooked-end steel fiber and straight steel fiber with lengths that were smaller than 10 mm. 

**Figure 9:**
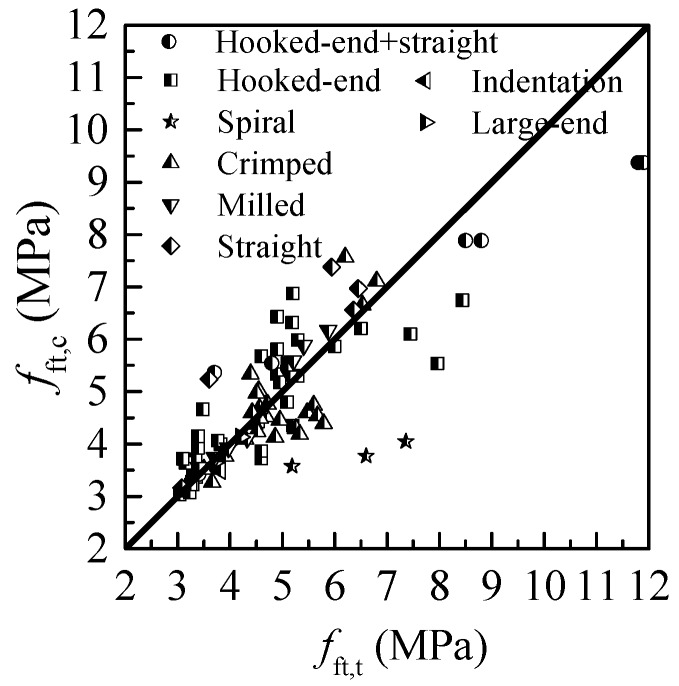
Comparison of calculated and measured values of tensile strength for SC-SFRC using Equations (6) and (7).

Figure 9 and Table 3 provide the detailed comparison of calculated tensile strength *f*_ft,c_ and tested tensile strength *f*_ft,t_ of SC-SFRC. The results show that Equation (6) with advised values of *α*_tb_*α*_te_ is suitable for predicting the tensile strength of SC-SFRC with the steel fibers of hooked-end, crimped, milled, indentation and large-end. Equation (6) with advised values of *α*_tb_*α*_te_ obviously underestimates the tensile strength of SC-SFRC with spiral steel fibers and overestimates that of SC-SFRC with straight steel fibers.

## 5. Conclusions

Based on the statistical analyses of test data for SC-SFRC, the following conclusions are drawn:

SC-SFRC has differences in the mix proportion compared with SCC. SC-SFRC usually has higher contents of binder paste and fine aggregate, higher sand ratio, lower content and smaller maximum particle size of coarse aggregates. Based on the content of coarse aggregates in the mix proportion, SC-SFRC could be divided into LC-type and NC-type. LC-type SC-SFRC has a significantly lower content of coarse aggregate and higher sand ratio compared with NC-type SC-SFRC. Most of the steel fibers used in SC-SFRC have lengths less than 37.5 mm, aspect ratios less than 70 and fiber factors no more than 0.45. The ratio of slump flows between SC-SFRC and the referenced SCC decreased with an increase in the fiber factor. 

Based on the calculation model of water-to-binder ratio in Chinese standard JGJ/T 55, suitable coefficients are advised for the mix proportion design of SC-SFRC. The calculation model of the designed tensile strength with advised coefficients according to Chinese standard JGT 472 is suitable for SC-SFRC with steel fibers of hooked-end, milled, indentation and large-end. The calculation model of the designed tensile strength with coefficients proposed in the authors’ previous study is suitable for SC-SFRC with crimped steel fibers. More adjustments in the mix proportion design of SC-SFRC, such as determining the dosages of binder materials and water and optimizing sand ratios, need to be further studied.

## Figures and Tables

**Table 1:** The range of results used for the experimental database.

Index		Minimum Value	Maximum Value
Water-to-binder ratio (*w*/*b*)		0.15	0.52
Water-cement ratio (*w/c*)		0.25	1.30
Coarse aggregate	Maximum particle size (MA) (mm)	8	25
	Apparent density (kg/m^3^)	2600	3170
Fine aggregate	Maximum particle size (MA) (mm)	2	5
	Fineness modulus	1.9	3.5
	Apparent density (kg/m^3^)	2590	2720
Sand ratio by mass *β*_s_ (%)		41	76
Cement density (kg/m^3^)		3090	3170
Steel fiber	Length *l*_f_ (mm)	6	60
	Aspect ratio *l*_f_/*d*_f_	15	120
	Volume fraction *ρ*_f_ (%)	0.08	1.79
Slump flow (SF) (mm)		500	830
The cubic compressive strength (*f*_cu_) (MPa)		20	120
The splitting tensile strength (*f*_ft_) (MPa)		3.0	12.4

**Table 2:** Proposed values for mortars with different fly-ash and *w*/*b* [65].

Percentage of Fly-Ash	0	10	20	30	40	50
Fly-ash of class I or superfine fly-ash; Fly-ash of class II with *w*/*b* ≤ 0.35	1	1.00–1.05	0.95–1.00	0.85–0.90	0.75–0.80	0.65–0.70
Fly-ash of class II with *w*/*b* > 0.35	1	0.90–0.95	0.80–0.85	0.70–0.75	0.60–0.65	-

**Table 3:** Comparison of *f*_ft,c_ and *f*_ft,t_ of SC-SFRC.

Fiber Type	Number of Tests Data	Mean Ratio of *f*_ft,c_/ *f*_ft,t_	Standard Deviation	Coefficient of Variation
Hooked-end	38	1.042	0.154	0.148
crimped	25	0.962	0.124	0.128
Milled	8	1.030	0.045	0.044
Straight	5	1.167	0.183	0.157
Spiral	3	0.603	0.075	0.124
Indentation	1	0.916	–	–
Large-end	1	0.984	–	–
Hooked-end & straight	8	1.011	0.219	0.216

## Appendix A

**Table A1:** Ranges of main parameters of SC-SFRC.

Ref. no	Cement Type	*N*	*w*/*b*	*w*/*c*	MA (mm)	*β*_s_ (%)	Steel Fiber				*f*_cu_ (MPa)	*f*_ft_ (MPa)	SF (mm)
							Type	*l*_f_ (mm)	*l*_f_/*d*_f_	*ρ*_f_ (%)			
[2]	CEM III 42.5N + CEM I 52.5R or/and CEM III 52.5A	16	0.30	0.47	16	68	Hooked-end	60	80	0.77	54	6.3	–
			0.30	0.47	16	68	Hooked-end	30	80	0.77	58	6.6	–
			0.30	0.47	16	68	Hooked-end	40	65	1.28	52	6.6	–
			0.30	0.47	16	68	Hooked-end	30	45	1.79	56	7.6	–
			0.32	0.46	8	70	Hooked-end	30	80	0.51	70	7.6	–
			0.32	0.46	8	70	Hooked-end	20	65	0.77	76	7.4	–
			0.31	0.44	16	68	Hooked-end	60	80	0.77	75	8.7	–
			0.31	0.44	16	68	Hooked-end	30	80	0.77	72	8.1	–
			0.31	0.44	16	68	Hooked-end	40	65	1.28	74	8.9	–
			0.31	0.44	16	68	Hooked-end	30	45	1.79	78	9.8	–
			0.31	0.44	16	68	Hooked-end	60	80	0.77	75	8.1	–
			0.31	0.44	16	68	Hooked-end	30	45	1.79	72	9.6	–
			0.28	0.38	16	68	Hooked-end	60	80	0.77	117	12.4	–
			0.30	0.47	16	68	Hooked-end	30	45	0.77	52	5.6	–
			0.30	0.47	16	68	Hooked-end	30	45	1.54	56	7.3	–
			0.28	0.38	16	68	Hooked-end	30	80	0.77	114	11.6	–
[9]	CEM II 42.5	9	0.34	0.49	15	50–52	Hooked-end	25	25	0.35–1.0	42–46	3.0–3.3	653–693
							Hooked-end	30	30	0.35–1.0	43–48	3.2–3.4	625–655
							Hooked-end	50	50	0.35–1.0	40–45	3.3–3.4	594–638
[10]	ASTM Type III	3	0.30	0.43	19	49–50	Hooked-end	60	80	0.5	100–110	7.5–8.4	700–785
[12]	P.O 42.5	6	0.31–0.34	0.61–0.67	20	50–57	Straight	30	60	0.3–1	35–69	2.5–5.3	665–680
[13]	P.II 52.5	7	0.32–0.36	0.46–0.57	16	46–47	Crimped	25	50	0.38–0.89	75–84	–	445–665
[14]	P.O.42.5R	3	0.22	0.31	20	46	straight	10	62.5	0.3–0.9	98–103	–	570–655
[15]	OPC #53	9	0.31	0.48	16	45–48	-	13	14	0.5–1.5	45–48	4.5–4.7	650–685
							-	23	25	0.5–1.5	46–48	4.6–4.7	590–655
							-	32	35	0.5–1.5	45–47	4.4–4.5	565–605
[16]	CEM II	4	0.31	0.40	15	–	Hooked-end	30	60	0.2–0.8	55–59	4.9–5.3	580–690
[17]	CEM I	5	0.23	0.27	10	–	screw-type	25	50	0.08–0.51	112–120	5.2–7.4	700–730
			0.22	0.31	10	45	Straight	10	67	0.31–0.61	98–103	–	630–655
[18]	P.O.42.5	3	0.30	0.38	20	48–49	dumbbell-shaped	20	50	0.6–0.8	79–82	–	635–680
[19]	P.O.42.5	1	0.32	0.45	20	49	Hooked-end	35	65	0.26	72	5.3	720
[22]	Type I PC	5	0.25–0.32	0.42–0.62	19	44–56	Hooked-end	30	60	0.51–1.02	55–85	–	395–645
			0.32	0.57	19	54	Hooked-end	50	100	0.51	60	–	620
[23]	OPC #53	3	0.23–0.36	0.37–0.58	20	52	Hooked-end	35	65	0.5–0.75	46–71	4.9–6.5	610–660
[26]	CEM I 42.5R	1	0.15	0.27	12	59	Hooked-end	60	80	0.38	62	–	720
[27]	ASTM Type I	3	0.40	0.904	19	60	Hooked-end	30	55	0.77	26	3.1	660
			0.36	0.82	19	–	Hooked-end&straight	–	–	0.77	30	3.4	630
			0.36	0.82	19	60	Straight	6	37.5	0.77	30	3.1	700
[28]	P.O 42.5	9	0.33	0.41–0.55	20	53–76	Hooked-end	35	65	0.6–1.2	45–61	3.7–6.4	650–700
[29]	CEM I42.5 N	6	0.34–0.39	0.5	16	61	Hooked-end&straight	30	60	0.4–0.7	41–44	–	500–600
[31]	CEM II 42.5	7	0.40–0.44	0.44–048	8	49	Hooked-end	35	65	0.64	49–62	2.9–5.1	450–850
[32]	CEM I42.5 R	4	0.21	0.39	13	59	Hooked-end	33	60	0.77	75–85	–	680–720
[33]	P.O 42.5	3	0.34	0.5	20	44	Crimped	15	40	0.5–1.25	54–63	–	650–685
[34]	CEM I 52.5R	2	0.36	0.47	18	55	Hooked-end	50	50	0.45	–	–	540
							Hooked-end	30	79	0.45	–	–	590
[35]	P.O 42.5	9	0.31–0.37	0.39–0.48	20	48	Hooked-end	35	44	0.2–0.6	35–60	–	610–705
[36]	P.O 42.5	7	0.35	0.50	10	49–50	Hooked-end	60	80	0.26–0.76	49–62	–	650–710
			0.33–0.35	0.46–0.50	10	48–50	Hooked-end	35	65	0.32–0.64	42–67	–	650–760
[37]	CEM I 42.5R	12	0.41	0.41	8	47–49	Crimped	35	28	0.5–1.5	69–77	–	740–805
							Crimped	50	25	0.5–1.5	75–79	–	720–795
[38]	P.C 32.5R	7	0.45	0.56	10	45	Hooked-end	35	65	0.25–0.51	35–36	–	420–580
	P.O 42.5R		0.35	0.5	10	51	Hooked-end	35	65	0.32–0.64	42–48	–	730–760
	P.II 52.5R		0.33	0.46	10	50	Hooked-end	35	65	0.32–0.64	65–67	–	650–700
[39]	–	5	0.22–0.41	0.25–0.41	10	51–63	Hooked-end	30	55	0.51–0.64	80	–	560–830
[40]	–	1	0.41	0.41	11	60	Hooked-end	50	50	0.64	61	–	570
[41]	CEM 42.5 R	1	0.34	0.34	–	–	Hooked-end	33	60	0.8	63	–	670
[42]	CEM II/A-L 42.5 R	1	0.34	0.40	10	–	Straight	11	27.5	0.65	45	–	650
[43]	ASTM Type I	3	0.32	0.71	10	48	Hooked-end	30	60	0.5–1.5	55–67	–	550–670
[44]	P.O 42.5	11	0.34	0.49	20	46–49	Mill-cut	32	40	0.3-1.2	40–45	5.2–6.2	650–685
[45]	OPC # 53	9	0.35	0.35	20	41	Straight	50	–	–	41–42	3.5–4.3	–
							Hooked-end	50	–	–	43–44	4.0–4.3	–
							Crimped	50	–	–	44–48	4.0–4.5	–
[46]	CEM I 52.5R	12	0.26	0.26	10	–	Double Hook-end	60	65	0.25–1.0	66–74	–	730–810
							Double Hook-end	60	65	0.25–1.0	66–71	–	765–830
							Single hooked-end	60	65	0.25-1.0	67–73	–	740–810
[47]	CEM I 42.5R	6	0.43	0.43	15	70	Hooked-end	30	45	0.5	68	–	740
							straight	6	37.5	0.5	67	–	760
							mixed			0.5	67	–	750
				0.85	15	70	Hooked-end	30	45	0.5	68	–	710
							straight	6	37.5	0.5	65	–	720
							mixed			0.5	69	–	700
[48]	CPIII 40	4	0.32	0.5	9.5	–	Hooked-end	35	65	1.0–1.5	–	–	–
							Hooked-end and straight	35/12	65/67	1.0–1.5	–	–	–
[49]	SLC	1	0.52	1.3	10	–	Hooked-end	60	80	0.38	50	4.5	670
[50]	CEM1 42.5R	2	0.24	0.45	12	–	Hooked-end	60	80	0.38–0.57	84–89	–	700
[51]	ASTM type I	22	0.41	0.77	12	65	Hooked-end	30	80	0.5–1.5	50–59	–	570–650
							Hooked-end	30	55	0.5–1.5	50–58	–	585–635
							4DHooked-end	60	65	0.5–1.5	55–60	–	535–635
							5DHooked-end	60	65	0.75–1.5	52–54	–	505–555
			0.29	0.36	12	63	Hooked-end	30	80	0.5–1.5	82–87	–	570–720
							4DHooked-end	60	65	0.75–1.5	88–89	–	595–750
							5DHooked-end	60	65	0.75–1.5	82–85	–	685–720
[52]	ASTM Type I	18	0.32	0.46	15	48.6	Hooked-end	30	55	0.26–0.77	73–90	–	600–750
						48.6	Hooked-end	60	80	0.26–0.77	65–86	–	530–720
						63.2	Hooked-end	30	55	0.26–0.77	79–85	–	640–755
						63.2	Hooked-end	60	80	0.26–0.77	73–85	–	600–750
						71.5	Hooked-end	30	55	0.26–0.77	77–83	–	680–765
						71.5	Hooked-end	60	80	0.26–0.77	75–81	–	660–760
[53]	ASTM Type II	8	0.48	0.48	12.5	60	Milled-cut	20.6	20	0.4–1.5	43–50	3.4–4.3	640–750
			0.38	0.42	12.5	60	Milled-cut	20.6	20	0.4–1.6	71–76	5.1–5.9	730–780
[54]	CEM II/B 42.5N	13	0.4	0.4	20	50	Crimped	35	58	0.3–1.2	48–53	4.3–5.6	650–800
							Crimped	40	67	0.3–1	51–53	4.9–5.7	650–795
							Crimped	50	83	0.3–1	52–53	5.3–5.8	630–750
[55]	Type II PC	6	0.23	0.37	10	52–53	Crimped	25	50	0.5–1.0	100–106	6.2–6.8	720–770
							straight	–	–	0.5–1.0	99–103	5.9–6.4	730–780
[56]	Type II PC	4	0.62	0.62	6	69	Hooked-end	35	65	0.19–0.77	29–33	–	580–770
[57]	CEM I 42.5R	9	0.44	0.44	16	46	Straight and crimped mixed	6/35	32/27	1–2	39–48	–	700–790
[58]	High Strength PC	9	0.32	0.5	13.2	64–65	Hooked-end	35	65	0.5–1	52–63	3.2–5.2	570–675
[59]	ASTM Type I	5	0.35	0.4	19	63	Hooked-end and straight mixed	35/6	55/37.5	0.77	44–59	3.6–5.1	615–695
[60]	P.O 42.5	6	0.31–0.32	0.44–0.46	16	53–57	Crimped	38.8	33.2	0.5–1.5	46–57	3.5–3.9	690–740
							Indentation	32.3	28.5	1	49	3.8	715
							Hooked-end	50.7	63.3	1	48	3.8	660
							Large-end	52.2	67.4	1	47	4.2	665
[61]	CEM II/A-LL 42.5R	1	0.29	0.44	12	54	Hooked-end	50	63	0.51	68	–	720

Note: ***N*** is the number of groups in references. A group of test data is a set of results tested from SC-SFRC with the same mix proportion and type and content of steel fiber in the reference.
